# Impact of COVID-19 Pandemic on University Students' Physical Activity Levels: An Early Systematic Review

**DOI:** 10.3389/fpsyg.2020.624567

**Published:** 2021-01-15

**Authors:** Alejandro López-Valenciano, David Suárez-Iglesias, Miguel A. Sanchez-Lastra, Carlos Ayán

**Affiliations:** ^1^Observatory of Healthy & Active Living of Spain Active Foundation, Centre for Sport Studies, King Juan Carlos University, Madrid, Spain; ^2^GO Fit LAB, Ingesport, Madrid, Spain; ^3^VALFIS Research Group, Institute of Biomedicine (IBIOMED), Faculty of Physical Activity and Sports Sciences, University of León, León, Spain; ^4^Department of Special Didactics, Faculty of Education and Sport Science, University of Vigo, Pontevedra, Spain; ^5^Well-Move Research Group, Galicia Sur Health Research Institute (IIS Galicia Sur), Department of Special Didactics, Faculty of Education and Sport Science, University of Vigo, Pontevedra, Spain

**Keywords:** COVID-19, physically active lifestyle, undergraduate students, college students, tertiary education, lockdown, confinement, quarantine

## Abstract

**Purpose:** This systematic review aimed to analyze the impact that the COVID-19 lockdown had on the amount of physical activity performed by university students.

**Materials and Methods**: A systematic electronic search for studies providing information regarding physical activity levels pre and during COVID-19 pandemic in university students was performed up to 20th October 2020 in the databases Cochrane Library, PubMed, SPORTDiscus, and Web of Science. The risk of bias of external validity quality of included studies was assessed by means of those the Newcastle-Ottawa Scale (NOS). The quality of the evidence for main outcomes was graded using the Grading of Recommendations Assessment, Development and Evaluation (GRADE) approach.

**Results and Conclusions**: A total of 10 studies were selected. Physical activity levels were assessed by means of questionnaires (10 studies) and accelerometer (1 study). Risk of bias was regarded as low and high in six and four investigations, respectively. The quality of evidence was downgraded to low. A significant reduction of physical activity levels were observed in 9 studies. Compared to pre-lockdown values, five studies showed a reduction of light/mild physical activity (walking) between 32.5 and 365.5%, while seven studies revealed a reduction of high/vigorous physical activity between 2.9 and 52.8%. Walking, moderate, vigorous, and total physical activity levels have been reduced during the COVID-19 pandemic confinements in university students of different countries. Despite of the reductions, those who met the current minimum PA recommendations before the lockdown generally met the recommendations also during the confinements.

## Introduction

The world is experiencing a life-threating situation due to the COVID-19 pandemic. By 14th October 2020, there have been 37.888.384 confirmed cases, including 1.081.868 deaths (World Health Organization, 2020a). We still do not have silver bullets or shortcuts, and the answer requires to use every single tool in the toolbox (World Health Organization, 2020b). To this purpose, one of the most important strategies is to reduce mixing of susceptible and infectious people through early ascertainment of cases or reduction of contact (i.e., social distancing; Lewnard and Lo, 2020), implementing measures such as quarantines and lockdowns, which have proven highly effective in controlling the spread of the disease (Baker et al., 2020). These extreme measures, nevertheless, not only have economic consequences (Bonaccorsi et al., 2020). Changes in lifestyle such as reduced physical activity (PA) and unhealthy diet (Ammar et al., 2020a), as well as compulsory measures such as social distance derived from the lockdowns, can also affect both the physical and the mental health of the population worldwide (Ammar et al., 2020b; Mattioli et al., 2020).

Physical inactivity is considered as another pandemic by itself (Hall et al., 2020). It is a major cause of non-communicable chronic diseases, responsible for more than three million premature deaths per year worldwide (Lee et al., 2012; Lim et al., 2012) and the conservatively estimated cost for the healthcare systems was $53.8 billion dollars in 2013 (Ding et al., 2016). Before the COVID-19 outbreak, globally, 23% of adults and 81% of adolescents (aged 11–17 years) did not meet the World Health Organization global recommendations on PA for health (World Health Organization, 2018), and the trend was that physical inactivity was not increasing, while it was time spent on sedentary behavior (Guthold et al., 2018; Du et al., 2019).

Previous studies have identified an increase in physical inactivity during the transition from adolescence to adulthood and throughout the college/university years (Bray and Born, 2004; Jung et al., 2008; Crombie et al., 2009; Pullman et al., 2009; Kwan et al., 2012). Pengpid et al. (2015) estimated that prevalence of physical inactivity among university students in 23 low, middle and high-income countries was 41%.

Social distancing and confinements have largely altered the lifestyle of university students, and it is not clear how the changes in the aforementioned factors are affecting the PA levels of this population. This review aimed to analyze if the PA levels of university students changed during the confinements and their adherence to the current global PA recommendations. Despite existing recommendations, suggesting several potential tactics (i.e. home-based exercise, dance, yoga) to keep active during the lockdown that are available to young populations (Chtourou et al., 2020), we hypothesized that total PA levels would be reduced due to the confinement.

## Methods

This systematic review was carried out following the Preferred Reporting Items for Systematic Reviews and Meta-Analysis (PRISMA) guidelines (Moher et al., 2009). The PRISMA checklist is presented in Appendix 1 (Supplementary Material).

### Search Strategy

A systematic computerized search was conducted up to 20th October 2020 in the databases Cochrane Library, PubMed, SPORTDiscus and Web of Science, following search terms included in Boolean search strategies: (coronavirus OR COVID-19 OR lockdown) AND (physical activity OR exercise OR activity) AND (university OR college OR student). Finally, the reference lists of the studies recovered were hand-searched to identify potentially eligible studies not captured by the electronic searches. Search strategies can be found in online Appendix 2 (Supplementary Material).

Two reviewers independently (AL-V and DS-I): (a) screened the title and abstract of each reference to locate potentially relevant studies, and once hard copies of the screened documents were obtained; (b) reviewed them in detail to identify articles that met the selection criteria. A third external reviewer (CA) was consulted to resolve discrepancies between reviewers in the studies selection.

### Study Selection

To be included in this systematic review studies had to fulfill the following criteria: (1) studies had to report PA levels pre and during COVID-19 pandemic in university students; (2) studies had to assess PA level through a valid and reliable tool; (3) studies had to be published in a peer-reviewed journal before 20th October 2020; (4) studies had to be written in English or Spanish. Literature reviews, abstracts, editorial commentaries, and letters to the editors were excluded.

### Risk of Bias and Quality of the Evidence

Two reviewers independently assessed the risk of bias of external validity quality of included studies using the “Newcastle-Ottawa Scale (NOS)” for cohort studies. The original NOS is a quality assessment tool for cohort and case-control studies which contains eight items categorized into three domains (selection, comparability and exposure) and uses a star rating system to indicate the quality of a study (one star for each item within the Selection and Outcome categories, and a maximum of two stars in Comparability category) (Wells et al., 2012). NOS scores categorized into three groups: very high risk of bias (0–3 NOS points), high risk of bias (4–6), and low risk of bias (7–9) (Lo et al., 2014).

The quality of the evidence for main outcomes was graded (high, moderate, low, or very low certainty) using the Grading of Recommendations Assessment, Development and Evaluation (GRADE) approach. Four different GRADE factors were used in this meta-analysis: risk of bias, inconsistency, indirectness and imprecision (Guyatt et al., 2011). The starting point was always the assumption that the pooled or overall result was of high quality. The quality of evidence was subsequently downgraded by one or two levels per factor to moderate, low, or very low when there is a risk of bias, inconsistency, imprecision or indirect results (Balshem et al., 2011).

In order to assess the inter-coder reliability of the coding process, two researchers coded all studies (including risk of bias and quality of the evidence assessment). The inconsistencies between the two coders were resolved by consensus, and when these were due to ambiguity in the coding book, this was corrected. As previously mentioned, any disagreement was resolved by mutual consent in consultation with a third reviewer.

## Results

### Descriptive Characteristics of the Studies

**Figure 1:**
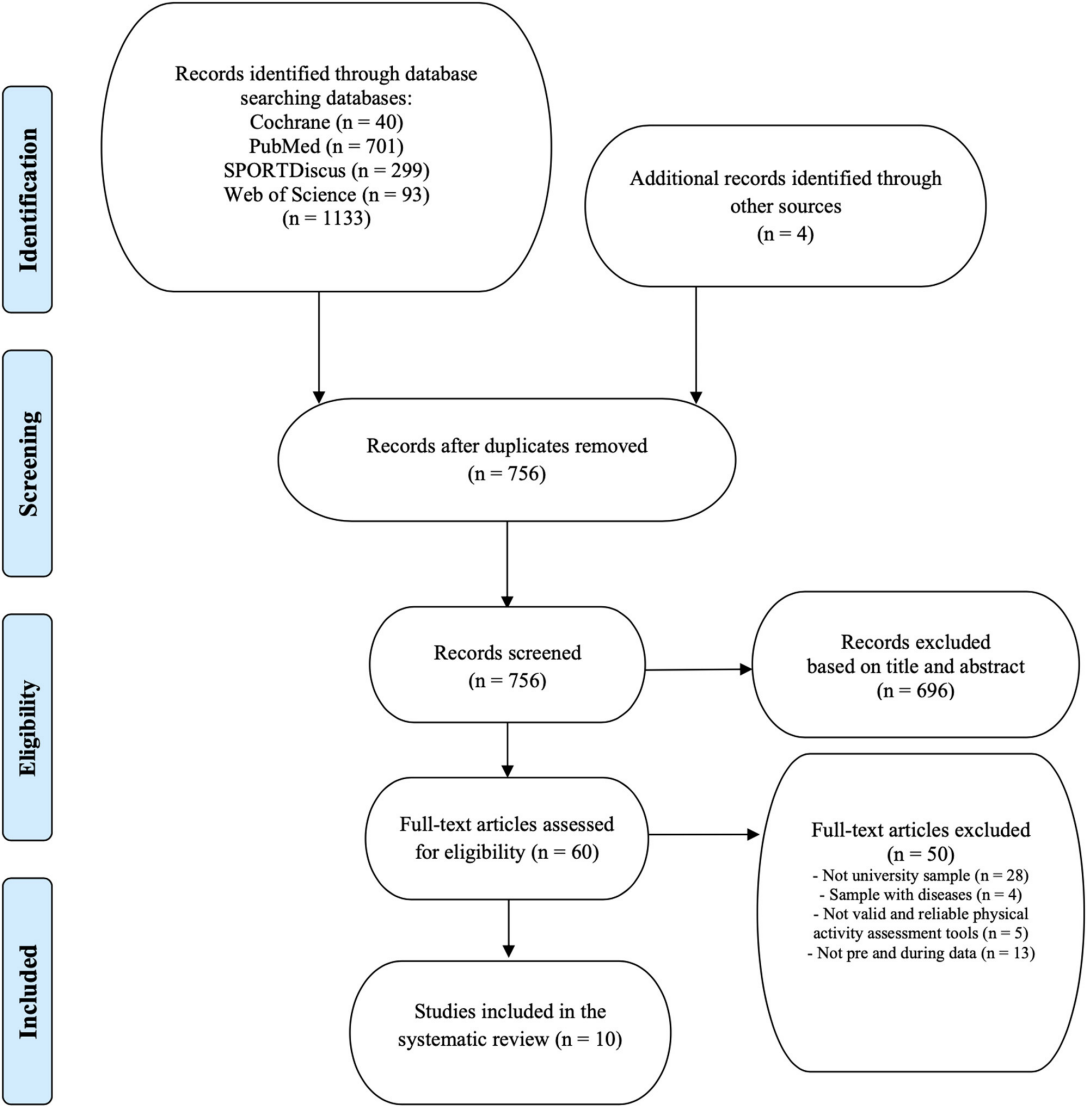
Systematic literature review process.

One thousand one hundred thirty-seven references were identified after search process in four databases, of which 10 (Ács et al., 2020; Barkley et al., 2020; Gallè et al., 2020; Gallo et al., 2020; Karuc et al., 2020; Maher et al., 2020; Romero-Blanco et al., 2020; Sañudo et al., 2020; Savage et al., 2020; Alarcón Meza and Hall-López, 2021) met the inclusion criteria. Figure 1 shows the flow chart of the selection process of the studies. The main characteristics of the studies included in this systematic review are presented in Table 1. Two studies were carried out in Spain, two in The United States of America, one in Australia, one in Croatia, one in England, one in Hungary, one in Italy and one in Mexico. The total sample size was larger than 3,500 university students.

**Table 1:** Characteristics of the included studies in the review.

**Study and Country**	**Design (type study/registration time/assessment)**	**Final sample**	**PA assessment tool**	**Severity of restricted internal movement by country**	**Levels of PA pre COVID-19 lockdown**	**Levels of PA during COVID-19 lockdown**	**Other results**
Ács et al. (2020) Hungary	Cross-sectional study April-end of May Q	*N* = 827 students of 10 faculties at the University of Pécs (25.3 ± 8.1 years old) ♂ = 182 ♀ = 645	IPAQ	National state of emergency. Universities were ordered to suspend in-person classes and switch to online eLearning courses	Walking, *min/week*: 342.6 ± 303.5 Moderate, *min/week*: 126.7 ± 213.7 Vigorous, *min/week*: 142.9 ± 195.7 Total PA, *min/week*: 609.8 ± 499.2	Walking, *min/week*: 162.5 ± 237.8 (↓52.6%) [*p* < 0.001] Moderate, *min/week*: 136.7 ± 220.6 (↑7.9%) [*p* = 0.170] Vigorous, *min/week*: 138.7 ± 180.5 (↓2.9%) [*p* = 0.484] Total PA, *min/week*: 435.4 ± 472.0 (↓28.6%) [*p* < 0.001]	Pre-lockdown: vigorous PA was higher among ♂ than ♀ (*p* = 0.047) No significant difference was found between genders in comparing total values of PA before and during COVID-19 (*p* = 0.532, *p* = 0.700, respectively)
Alarcón Meza and Hall-López (2021) Mexico	Cross-sectional study DNS Q	*N* = 32 students of the Faculty of Sports of the Autonomous University of Baja California (21.4 ± 3.6 years old) ♂ = 17 ♀ = 15	IPAQ	–	Low, *% of participants*: 3.6% Moderate, *% of participants*: 5.2% High, *% of participants*: 91.2 Weekly energy expenditure of PA, *MET-min/week*: 6,473	Low, *% of participants*: 10.9 (↑7.3%) Moderate, *% of participants*: 6.8 (↑1.6%) High, *% of participants*: 82.3 (↓8.9%) Weekly energy expenditure PA, *MET- min/week*: 4,297 (↓33.6%) [*p* = 0.005]	
Barkley et al. (2020) USA	Cross-sectional study May 18–June 3 Q	*N* = 100 undergraduate students (26.9 ± 8.9 years old)	GSLTPAQ^a^	20 March, the campus (including all fitness facilities) was closed soon thereafter and all students were sent home 22 March, the university's home state issued a “stay at home” order	Mild, *Godin score*: 16.3 ± 22.6 Moderate, *Godin score*: 15.0 ± 15.7 Vigorous, *Godin score*: 16.0 ± 22.1 Total PA, *Godin score*: 47.2 ± 40.2	Mild, *Godin score*: 10.8 ± 12.9 (↓32.5%) [*p* = 0.015] Moderate, *Godin score*: 12.9 ± 12.4 (↓14%) Vigorous, *Godin score*: 14.0 ± 17.9 (↓12.5%) Total PA, *Godin score*: 37.7 ± 30.7 (↓20.1%)	
Gallè et al. (2020) Italy	Cross-sectional study Last three weeks of May Q	*N* = 1,430 students from three Italian universities (22.9 ± 3.5 years old) ♂ = 494 ♀ = 936	IPAQ	Localized and national lockdown. Grocery shopping and walking pets were the only activities allowed	Walking, *min/week*: 480 Moderate, *min/week*: 199.3 Vigorous, *min/week*: 138.6 Total PA, *min/week*: 520 ± 820	Walking, *min/week*: 114.5 (↓365.5%) [*p* < 0.05] Moderate, *min/week*: 148.1 (↓51.2%) [*p* < 0.05] Vigorous, *min/week*: 108.3 (↓30.3%) [*p* < 0.05] Total PA, *min/week*: 270 ± 340 (↓50%) [*p* < 0.0001]	During lockdown: 639 participants (44.7%) remained sufficiently active. Being younger than 22 years old, female, and previously active, attending the universities of Naples and Rome, and having at least one graduate parent were associated with the achievement of recommended levels of PA
Gallo et al. (2020) Australia	Longitudinal study Pre: March 19–21 2018 (T_1_), March 25–27 2019 (T_2_), and March 29-April 3 2020 (T_3_) During: May 12–26 (T_4_) Q	*N* = 509 students from the University of Queensland (22.5 ± 0.08 years old) ♂ = 214 ♀ = 295	The Active Australia Survey	Localized lockdown 23 March, all but essential services were shut down and universities transitioned all undergraduate learning online 30 March, people were only allowed to leave their homes for work (in an essential service), or to purchase food, receive or provide medical care, or exercise	♂ Walking, *min/week* (median ± IQR): T_1_: ~150 ±~100; ~250 T_2_: ~130 ±~100; ~220 ♂ Vigorous, *min/week* (median ± IQR): T_1_: ~245 ±~110; ~400 T_2_: ~135 ±~100; ~300 ♀ Walking, *min/week* (median ± IQR): T_1_: ~125 ±~100; ~90 T_2_: ~125 ±~100; ~200 ♀ Vigorous, *min/week* (median ± IQR): T_1_: ~120 ±~60; ~220 T_2_: ~120 ±~55; ~215	♂ Walking, *min/week* (median ± IQR): T_3_: ~75 ±~60; ~145 (T_1_: ↓50%; T_2_: ↓42.3%) [*p* < 0.0001 between T_3_ and T_1_] ♂ Vigorous, *min/week* (median ± IQR): T_3_: 100 ±~70; ~220 (T_1_: ↓59.2%; T_2_: ↓25.9%) [*p* < 0.0001 between T_3_ and T_1_] ♀ Walking, *min/week* (median ± IQR): T_3_: ~100 ± ~50; ~185 (T_1_: ↓20%; T_2_: ↓20%) [*p* < 0.05 between T_3_ and T_2_] ♀ Vigorous, *min/week* (median ± IQR): T_3_: ~90 ± ~55; ~145 (T_1_: ↓25%; T_2_: ↓25%)	♂ Time spent walking: T_3_ < T_2_ (↓52.5 min) [*p* < 0.05] T_3_ < T_1_ (↓87.5 min) [*p* < 0.0001] ♂ Time spent in vigorous activity [*p* < 0.0001]: T_3_ < T_2_ (↓60 min) [*p* < 0.05] T_3_ < T_1_ (↓150 min) [*p* < 0.0001] ♀ Time spent walking: T_3_ < T_2_ (↓30 min) [*p* < 0.05] T_3_ < T_1_ (↓30 min) [*p* < 0.068] ♂ No differences in time spent in vigorous activity between the time points of the study PA levels T_4_ vs. T_3_: No change for the majority of ♂ PA levels T_4_ vs. T_3_: increased for >40% of ♀
Karuc et al. (2020) Croatia	Longitudinal survey design April 24–May 8 Q	*N* = 91 university students ♂ = 32 (21.5 ± 0.3 years old) ♀ = 59 (21.6 ± 0.4 years old)	SHAPES	National lockdown. Government measures to restrict gathering in public places and parks, suspend public transportation, and close institutions. All social gatherings, work in retail and services including sports activities were prohibited	♂ MVPA, *min/day* (median ± IQR): 135 ± 127.5 ♀ MVPA, *min/day* (median ± IQR): 120 ± 227.1	♂ MVPA, *min/day* (median ± IQR): 85.7 ± 56.8 (↓36.5%; ↓57.7 *min/day* [*p* = 0.006]) ♀ MVPA, *min/day* (median ± IQR): 64.3 ± 75.0 (↓46.4%; 64.8 *min/day* [*p* < 0.0001])	♂ Same PA levels: 31% ♂ Increased PA levels: 19% ♂ Decreased PA levels: 50% ♀ Same PA levels: 25% ♀ Increased PA levels: 19% ♀ Decreased PA levels: 56%
Maher et al. (2020) USA	Cross-sectional study Pre: January 21–March 11 During: April 17–May 5 Q	*N* = 107 undergraduate kinesiology students (21.7 ± 2.6 years old)	IPAQ-SF	March 13, campus closure March 25, executive orders banning mass gatherings and closure of non-essential businesses for the state March 30–May 8, mandatory stay-at-home orders for the state	MVPA, *min/week*: 424.6 ± 372.0	MVPA, *min/week*: 324.7 ± 316.6 (↓23.5%) [*p* = 0.02]	
Romero-Blanco et al. (2020) Spain	Cross-sectional study Pre: January 15–30 During: April 1–15 Q	*N* = 213 health sciences students (20.5 ± 4.5 years old) ♂ = 41 ♀ = 172	IPAQ-SF	Localized and national lockdown March-April, prohibition on going outside to engage in sporting or social activities	Moderate, *min/week*: 42.8 ± 48.4 Vigorous, *min/week*: 28.5 ± 54.1 Total PA, *min/week*: 223.3 ± 305.5 ♂ Total PA, *min/week*: 226.5 ± 250.1 min/week ♀ Total PA, *min/week*: 222.5 ± 317.9	Moderate, *min/week*: 47.7 ± 50.8 (↑4.7%) [*p* = 0.353] Vigorous, *min/week*: 30.6 ± 30.9 (↑7.4%) [*p* = 0.07] Total PA, *min/week*: 383.2 ± 438.9 (↑71.6%) [*p* < 0.001] ♂ Total PA, *min/week*: 279.9 ± 446.9 (↑23.6%) [*p* = 0.339] ♀ Total PA, *min/week*: 407.8 ± 404.8 (↑83.3%) [*p* < 0.001]	
Sañudo et al. (2020) Spain	Longitudinal survey design Pre: one week in February During: March 24–3 April Q and accelerometer	*N* = 20 university students (22.6 ± 3.4 years old) ♂ = 11 ♀ = 9	IPAQ Wristband	Localized and national lockdownMarch-April, prohibition on going outside to engage in sporting or social activities	Walking, *min/week*: 362 ± 262 Moderate, *min/week*: 441 ± 487 Moderate-to-vigorous, *min/week*: 797 ± 822 Vigorous, *min/week*: 356 ± 381 min/week Objectively measured PA, *steps/day*: 8,525 ± 3,597	Walking, *min/week*: 27 ± 47 (↓92.5%) [*p* < 0.0001] Moderate, *min/week*: 178 ± 155 (↓59.7%) [*p* = 0.028] Moderate-to-vigorous PA, *min/week*: 346 ± 341 (↓56.6%) [0.005] Vigorous, *min/week*: 168 ± 228 (↓52.8%) [*p* = 0.006] Objectively measured PA, *steps/day*: 2,754 ± 1,724 (↓67.7%) [*p* < 0.0001]	Participants meeting the PA guidelines (WHO): 84% at pre-lockdown, 74% during lockdown
Savage et al. (2020) England	Longitudinal cohort study Pre: October 14–20 2019 (T_1_), January 28-February 3 2020 (T_2_) During: March 20–26 March (T_3_, 1st week of lockdown), 27 April 27-May 3 (T_4_, 5th week of lockdown) Q	*N* = 214 students from East Midlands university (20 years old) ♂ = 60 ♀ = 154	EVS	People in the United Kingdom were required to stay at home as much as possible and were only allowed to leave once per day for exercise	♂ MVPA, *min/week*: T_1:_ 296 ± 254 ♀ MVPA, *min/week*: T_1_: 231 ± 232	♂ MVPA, *min/week*: T_4_: 220 ± 252 (↓25.7%) ♀ MVPA, *min/*week: T_4_: 222 ± 208 (↓3.9%)	At all-time points: average MVPA >150 min/week During T_3_-T_4_: ↓28 min/week of moderate to vigorous PA (on average) The reduction in PA was more pronounced in ♂ than ♀

*–, Not information available; min, minutes; DNS, date note specified; EVS, Exercise Vital Sign questionnaire; GSLTPAQ, Godin-Shephard Leisure-Time Physical Activity Questionnaire; IPAQ, International Physical Activity Questionnaire; IPAQ-SF, International Physical Activity Questionnaire-Short Form; SHAPES, School Health Action, Planning, and Evaluation System questionnaire; MET, metabolic equivalent; MVPA, moderate-vigorous physical activity; PA, physical activity; Q, questionnaire/survey*.

a *A score for each intensity is calculate using the following equations: times per week participating in strenuous × 9, moderate × 5, mild × 3. Each of these individual scores was then summed for a total physical activity score. ♀, female; ♂, male; ↑, increase; ↓, decrease/decline*.

Six out of ten studies used International Physical Activity Questionnaire (IPAQ) as tool to assess the level of PA performed by university students, while the Godin physical activity questionnaire, the Active Australia Survey, the School Health Action, Planning, and Evaluation System (SHAPES) questionnaire and Exercise vital sign (EVS) questionnaire were used in the rest of studies. Only one study (Sañudo et al., 2020) used an objective tool to assess PA (accelerometer). Regarding the level of the lockdown by country, most of the studies (Alarcón-Meza's study did not indicate the country's measures regarding PA during lockdown), reflected that national lockdown included a restriction for outdoor PA.

With regards to the reporting risk of bias of the studies, NOS scale showed that six studies had low risk of bias, while four studies got 6 stars, so they show a high risk of bias. The quality of evidence according to GRADE was downgraded to low (risk of bias, and indirectness). The detailed data for NOS and GRADE scales are presented in Tables 2, 3, respectively.

**Table 2:** Risk of bias assessment of the studies (Newcastle-Ottawa scale).

**Study**	**Criteria for assessing risk of bias**								
	**1**	**2**	**3**	**4**	**5**	**6**	**7**	**8**	**Total**
Ács et al. (2020)	^*^	^*^	^*^		^*^	^*^	^*^		6
Alarcón Meza and Hall-López (2021)	^*^	^*^	^*^		^*^^*^	^*^	^*^		7
Barkley et al. (2020)	^*^	^*^	^*^		^*^	^*^	^*^		6
Gallè et al. (2020)	^*^	^*^	^*^		^*^	^*^	^*^		6
Gallo et al. (2020)	^*^	^*^	^*^		^*^	^*^	^*^	^*^	7
Karuc et al. (2020)	^*^	^*^	^*^		^*^	^*^	^*^		6
Maher et al. (2020)	^*^	^*^	^*^		^*^	^*^	^*^	^*^	7
Romero-Blanco et al. (2020)	^*^	^*^	^*^		^*^	^*^	^*^	^*^	7
Sañudo et al. (2020)	^*^	^*^	^*^		^*^^*^	^*^	^*^	^*^	8
Savage et al. (2020)	^*^	^*^	^*^		^*^	^*^	^*^	^*^	7

*Criteria for assessing risk of bias: (1) representativeness of the exposed cohort; (2) selection of the non-exposed cohort; (3) ascertainment of exposure; (4) demonstration that outcome of interest was not present at start of study; (5) comparability of cohorts on the basis of the design or analysis (A maximum of two stars can be allotted in this category); (6) assessment. of outcome; (7) was follow-up long enough for outcomes to occur; (8) adequacy of follow-up of cohorts*.

* *Star(s) awarded for each criterion*.

**Table 3:** Summary of findings (GRADE).

**N**°** of studies**	**Certainty assessment**						**Certainty**
	**Study design**	**Risk of bias**	**Inconsistency**	**Indirectness**	**Imprecision**	**Other considerations**	
**Physical activity level pre-during COVID lockdown**							
10	Observational studies	Serious^a^	Not serious	Serious^b^	Not serious	Very strong association	⊕⊕◯◯ LOW

a *Four studies reported high risk of bias (assessed with the Newcastle Ottawa Scale)*.

b *The information provided by two studies does not answer completely the main question about levels of physical activity. These circles represent the degree of certainty of the variable analysed on the GRADE scale. A circle with a “+” symbol inside represents a very low certainty of this variable, two circles with the “+” symbol inside represent low certainty, three circles with the “+” symbol indicate moderate certainty and the four circles with the symbol “+” represent a high certainty of this variable*.

### Physical Activity Levels

Nine out of the ten studies included in the systematic review showed significant decreases in PA levels during lockdown, both in questionnaires as accelerometers. Surprisingly, one study (Romero-Blanco et al., 2020) showed significant increases in PA levels among university students during lockdown. Romero-Blanco et al. (2020) showed that Health Sciences university students performed significantly higher minutes/week of total PA (+71.6%) and vigorous PA (+7.4%), both males (+ 83.3%) as females (+ 23.6%). On the other hand, Sañudo et al. (2020) showed an objective reduction in PA with 67.7% fewer steps per day during the lockdown. In the same line, five studies showed a reduction of light/mild PA (walking) between 32.5 and 365.5% compared to the period prior to confinement (Ács et al., 2020; Barkley et al., 2020; Gallè et al., 2020; Gallo et al., 2020; Sañudo et al., 2020), three studies found a decrease in moderate PA levels (from 14–59.7%) (Barkley et al., 2020; Gallè et al., 2020; Sañudo et al., 2020) and four studies in moderate-to-vigorous PA (MVPA) (from 3.9–56.6%) (Karuc et al., 2020; Maher et al., 2020; Sañudo et al., 2020; Savage et al., 2020). Finally, seven studies also revealed a reduction of high/vigorous PA between 2.9 and 52.8% compared to pre-lockdown (Ács et al., 2020; Barkley et al., 2020; Gallè et al., 2020; Gallo et al., 2020; Romero-Blanco et al., 2020; Sañudo et al., 2020; Alarcón Meza and Hall-López, 2021) and two studies showed a decrease of total PA (28.6 and 50%) (Ács et al., 2020; Gallè et al., 2020). Regarding gender differences, two studies found a higher reduction of walking, vigorous and MVPA in males (Gallo et al., 2020; Savage et al., 2020), while only one study showed a higher reduction in females (Karuc et al., 2020). Otherwise, Romero-Blanco et al. (2020) found that women had performed more PA (min/week) than male during lockdown. The main results of the studies included in this systematic review are presented in Table 1. According to the reported data, those students who met the PA recommendations before the confinement took place, were still classified as physically active during the lockdown period.

## Discussion

This review aimed to analyze if PA levels of university students changed during the confinements in different countries. Our results are of interest from a public health perspective to the purpose of addressing the impact of the confinements on health-related habits such as PA and how we could help to reduce it and its derived problems.

We found that total of nine out of the ten included studies reported significant decreases in PA levels during the confinements. These results are in line with the findings from previous studies in both adults and children. Castañeda-Babarro et al. (2020) reported significant decreases in self-reported vigorous PA and walking time of 16.8 and 58.2%, respectively; whereas time spent in sedentary behavior increased during the confinement in Spain. The student group (from children to university students) showed the highest decrease in moderate, vigorous, and waking activities. In the mini-review from Arora and Grey (2020) the authors reported that increased social isolation is associated with higher rates of physical inactivity and sedentarism in adults. Dunton et al. (2020) reported that the COVID-19 pandemic has also negatively affected the PA levels of children living in the United States.

University students generally reduce its PA levels compared to their childhood. Factors affecting the decline of PA levels during this life stage include changes in psychosocial aspects and residency (i.e. distance to the university; Van Dyck et al., 2015) and greater time demands, such as work and class time (Calestine et al., 2017). Our findings expand this previous knowledge by suggesting that the reduction in total PA levels has been exacerbated during the confinements.

This finding is important for two main aspects. First, because it has been reported that the confinements developed to fight COVID-19 have increased mental health problems in both adults (Guo et al., 2020; Holingue et al., 2020; van Tilburg et al., 2020) and young populations (Arora and Grey, 2020; Jiao et al., 2020; Savage et al., 2020). Our results confirm that PA levels were generally reduced during the lockdowns compared to the previous situation. Efforts should be made to increase PA levels in this situation not only for the sake of physical health but also psychological well-being. Furthermore, incentivizing a routine through daily at-home PA could help maintaining a certain sense of routine and organization, helping to maintain mental health during the lockdown and also facilitate the routine back to university (Burtscher et al., 2020). Second, because it is well-known that sedentary behavior and insufficient PA patterns in childhood are likely to persist into adulthood, increasing the risk of major health complications (i.e. being overweight or obese, type II diabetes or hypertension; 2018 Physical Activity Guidelines Advisory Committee, 2018) and university students are transitioning within these two life stages.

Another interesting finding from our review is that, generally, those that were sufficiently active before the confinement (i.e., achieving current minimum recommendations for adults of at least 150 min per week of MVPA; U.S. Department of Health Human Services, 2018), were also sufficiently active during the lockdown, despite the reduction in PA levels. This finding, which has been reported also in adults (Castañeda-Babarro et al., 2020), could suggest that achieving the recommendations on MVPA help in creating a stronger habit of being physically active, which seemed not to be affected to a large extent by the confinements imposed due to COVID-19.

Our study has some strengths and limitations than need to be considered. First, while we included more than 3.500 university students from eight different countries, the pandemic affected differently each country and the level of restrictions may not be generalizable to other countries that were not represented in our review. Second, while we had some studies using direct measurements of PA, data were mostly collected from self-report, which is susceptible to cognitive bias. Third, we included six studies with low risk of bias following our methodological quality assessment, but the other 4 were considered to present high risk of bias. Fourth, a meta-analysis was not performed due to the heterogeneity of measurement tools, analyses and populations in the included studies, as well as in their methodological quality. Fifth, the heterogeneity in the analyses carried out in these studies did not allow to draw firm conclusions on how PA levels were differently affected for men and women. Finally, we included people from generally high-income countries.

Taken together, our findings suggest a decrease in PA levels from before to during the COVID-19 outbreak in university students of Australia, Croatia, England, Hungary, Italy, Mexico, Spain, and USA. In times of pandemic crisis, government and university leaders across these countries need to implement measures and advice to encourage this population to increase and maintain adequate levels of PA, as recently suggested by the WHO (Bull et al., 2020). In this context, a set of practical recommendations on how to be active outdoors and indoors during the current and ongoing COVID-19 pandemic can be applied to university students (Bentlage et al., 2020; Ammar et al., 2021). Physical activity programs, individually tailored to the participant's fitness level, should be developed. These programs could be delivered through gamification, communication and interactive coaching technologies (Ammar et al., 2021). For instance, group-based interventions using active videogames seem to be a motivating, enjoyable easy-to-use strategy for reducing social isolation among younger age groups (Viana and de Lira, 2020). This is of particular concern given the experience of loneliness tends to be most common in young adults (Beam and Kim, 2020). However, professional physical guidance, especially in the context of online PA sessions, is needed for university students (Deng et al., 2020). Hence, the work of exercise professionals would be essential to ensure that PA programs are properly designed, monitored and implemented, which is of most importance to guarantee safety and efficacy of exercise training and long-term PA adherence (Natalucci et al., 2020).

## Conclusion

Walking, moderate, vigorous, and total PA levels have been reduced during the COVID-19 pandemic confinements in university students of different countries. Despite of the reductions, those who met the current minimum PA recommendations before, generally met the recommendations also during the confinements.

## Data Availability Statement

The original contributions generated for the study are included in the article/Supplementary Material, further inquiries can be directed to the corresponding author/s.

## Author Contributions

DS-I and CA contributed in the conception and design of the study. AL-V and DS-I took part in the acquisition and analysis of data. AL-V, MAS-L, and CA contributed drafting the article. DS-I, AL-V, and CA approved the last version to be published. All authors were involved in interpretation of the data. All authors critically revised the article for important intellectual content.

## Conflict of Interest

The authors declare that the research was conducted in the absence of any commercial or financial relationships that could be construed as a potential conflict of interest.
