# Supplementary material for: Impact of COVID-19 Pandemic on University Students' Physical Activity Levels: An Early Systematic Review
Source: Front Psychol. 2021 Jan 15;11:624567. doi: 10.3389/fpsyg.2020.624567 (PMC7845570; doi:10.3389/fpsyg.2020.624567)
Supplement: Supplementary file 2 [file Table_2.DOCX]

# Appendix 2. Search strategies.

**Search strategy in PubMed - 120 results**

#1 ("COVID-19"[Title/Abstract] OR "coronavirus"[Title/Abstract] OR "lockdown"[Title/Abstract]) AND ("sedentary lyfestyle"[Title/Abstract] OR "sedentary"[Title/Abstract] OR "sedentary behavior"[Title/Abstract] OR "inactivity"[Title/Abstract])

#2 #1 Filters: Published up to 21^st^ October 2020.

**Search strategy in the Cochrane Central Register of Controlled Trials – 21 results**

#1 (coronavirus OR covid-19 OR lockdown) :ti,ab,kw AND (sedentary lifestyle OR sedentary behavior OR sedentary OR inactivity) :ti,ab,kw (Word variations have been searched)

#2 Filters: Published up to 21^st^ October 2020.

**Search strategy in Web of Science - 34 results**

#1 TI=(coronavirus OR covid-19 OR lockdown) AND TI=( sedentary lifestyle or sedentary behavior or sedentary or inactivity)

#2 Filters: Year to date (21^st^ October 2020)

**Search strategy in Sportdiscus - 60 results**

#1 TX (coronavirus or covid-19 or lockdown) AND TX (sedentary lifestyle or sedentary behavior or sedentary or inactivity)

#2 Filters: Published up to 21^st^ October 2020.
